# Evaluation of the therapeutic regimen in COVID-19 in transplant patients: where do immunomodulatory and antivirals stand?

**DOI:** 10.1186/s12985-021-01700-2

**Published:** 2021-11-22

**Authors:** Mojtaba Shafiekhani, Farbod Shahabinezhad, Tahmoores Niknam, Seyed Ahmad Tara, Elham Haem, Parviz Mardani, Zahra Zare, Sedigheh Jafarian, Khatereh Mirzad Jahromi, Sara Arabsheybani, Yalda Sadat Moeini, Jalile Alavi, Seyed Soroush Jalali, Maryam Salimi, Reza Shahriarirad, Seyed Ali Malekhosseini

**Affiliations:** 1 Shiraz Transplant Research Center, Shiraz University of Medical Sciences, Shiraz, Iran; 2 Shiraz Transplant Center, Abu-Ali Sina Hospital, Shiraz University of Medical Sciences, Shiraz, Iran; 3 Department of Clinical Pharmacy, Faculty of Pharmacy, Shiraz University of Medical Sciences, Shiraz, Iran; 4 Department of Biostatistics, School of Medicine, Shiraz University of Medical Sciences, Shiraz, Iran; 5 Thoracic and Vascular Surgery Research Center, Shiraz University of Medical Sciences, Shiraz, Iran; 6 Department of Surgery, Shiraz University of Medical Sciences, Shiraz, Iran; 7 Student Research Committee, Shiraz University of Medical Sciences, Shiraz, Iran

**Keywords:** COVID-19, Coronavirus disease 2019, Transplant, Tocilizumab, Remdesivir

## Abstract

**Background:**

The management of COVID-19 in organ transplant recipients is among the most imperative, yet less discussed, issues based on their immunocompromised status along with their vast post-transplant medication regimens. No conclusive study has been published to evaluate proper anti-viral and immunomodulator medications effect in treating COVID-19 patients to this date.

**Method:**

This retrospective study was conducted in Shiraz Transplant Hospital, Iran from March 2020 to May 2021 and included COVID-19 diagnosed patients based on SARS-CoV-2 RT-PCR positive test who had been hospitalized for at least 48 h before enrolling in the study. Clinical and demographic information of patients, along with their treatment course and the medication used were evaluated and analyzed using multiple regression analysis.

**Results:**

A total of 245 patients with a mean age of 49.59 years were included with a mortality rate of 8.16%. The administration of Remdesivir as an anti-viral drug (*P* value < 0.001) and Tocilizumab as an immunomodulator drug (*P* value < 0.001) could reduce the hospitalization period in the hospital and the intensive care unit, as well as the mortality rates significantly. Meanwhile, the patients treated with Lopinavir/Ritonavir experienced a lower chance of survival (OR < 1, *P* value = 0.04). No significant difference was observed between various therapeutic regimens in clinical complications such as bacterial coinfections, cardiovascular and gastrointestinal adverse reactions, and liver or kidney dysfunctions.

**Conclusion:**

The administration of Remdesivir as an anti-viral and Tocilizumab as an immunomodulatory drug in solid-organ transplant recipients could be promising treatments of choice to manage COVID-19.

## Introduction

Coronavirus disease (COVID-19), which is caused by the severe acute respiratory syndrome coronavirus 2 (SARS-CoV-2), has been altered to a major challenging health care system around the world since March 11, 2020, when the World Health Organization (WHO) confirmed COVID-19 as a pandemic [1–5]. According to WHO, up until August 12th, 2021, at least 204,644,849 cases of COVID-19 have been reported, of which at least 4,323,139 deaths have been caused by this disease, globally [6].

Clinical presentation in the general population mostly consists of constitutional symptoms; however, some cases can present with severe and potentially fatal conditions such as multiple organ failure, acute respiratory distress syndrome, pulmonary edema, and pneumonia, causing diagnosis and management dilemmas [7–12]. Based on the literature, a reduction in the absolute value of the lymphocytes was reported in most patients, indicating that the virus may mainly act on the lymphocytes, especially T-cells [12]. Consequently, patients with impaired or suppressed T-cells' function and number may be at a higher risk of presenting the severe form of the disease.

Regarding the impaired immune system from both underlying disease and treatment, immunocompromised patients, such as solid-organ transplant (SOT) recipients, are at risk of more severe respiratory virus infection and higher rates of bacterial and fungal superinfections compared with their immunocompetent counterparts [13]. A study in France reported that patients with kidney transplants demonstrated a high risk of mortality due to COVID-19 [14]. However, some studies have mentioned no statistically significant difference between the mortalities of transplant recipients and other COVID-19 patients [15, 16]. Therefore, it is suggested that policymakers should urgently ensure the integration of such risk factors on response operations against COVID-19.

Globally, vaccination efforts are being made against COVID-19 [17]. However, the slow rate of vaccination in the developing countries and the emergence of new variants, which could compromise vaccines’ efficacy, and also the lower efficacy of vaccines in the patients who take immunosuppressive treatments, still make the progress of managing this disease ineligible [18–22]. Therefore, it is critical to harness the experiences of managing and treatment of this disease to achieve better care for infected patients in the future.

Meanwhile, managing transplant recipients infected with SARS-CoV-2 is of great importance, since not only the reduction or discontinuation of immunosuppressive drugs would cause transplant rejection, but also increasing the immunosuppression rate would result in higher disease progression and a poor prognosis in the patient [23]. This makes it clear that a balance should be achieved in choosing immunosuppressive drugs, while also adjusting them is highly critical in transplant recipients. Keeping in mind that after seventeen months from the announcement of the SARS-CoV-2 outbreak, various pharmacological treatments were evaluated in this area from the early days up until now. As time passed, some of them were dismissed while several are still clinically used [24]. Many factors are considered in the management of COVID-19 among SOT recipients. Among these factors include the unknown role of immunomodulator drugs such as corticosteroids and interleukin-6 antagonists in COVID-19 treatment. Another factor worth exploring is the pharmaceutical phenomenon of both the COVID-19 and transplantation aspect, including polypharmacy in SOT recipients along with potential drug-drug interactions of the immunosuppressive medications with other therapeutic agents used in COVID-19 management, such as anti-viral drugs. These dilemmas encouraged us to evaluate and review the clinical outcomes of administrating different anti-viral and immunomodulatory drugs in hospitalized transplant patients in one of the largest transplant centers in Asia and the world.

## Patients and method

This retrospective study was conducted in Shiraz Transplant Center, Shiraz, Iran, affiliated to Shiraz University of Medical Sciences, as one of the largest centers for solid organ transplantation worldwide. During the COVID-19 pandemic period, many protocols were applied to control the rate of infection, such as reducing the number of admissions, reducing the number of surgeries and reducing the number of transplants compared to the same as last year, which these protocols have been mention in our previous report [25]. This study was approved by the ethics committee of Shiraz University of Medical Sciences (Ethical Code: IR.SUMS.REC.1399.398). The study timeline was during a period from March 2020 to May 2021 in which all transplant recipients diagnosed with COVID-19 were enrolled based on SARS-CoV-2 positive real-time polymerase chain reaction (RT-PCR) with hospitalization for more than 48 h. Demographic information of the patients along with the course of disease and array of signs and symptoms, medical history, clinical and radiographic findings, length of hospital stay (both in the ICU and ward), clinical outcome, and management of all patients were extracted from the medical records of the patients and evaluated accordingly. The patient’s severity was based on the WHO criteria for the clinical management of COVID-19 [26]. Also, the patient's admission was based on the joint discretion of an infectious specialist and transplant surgeon, for patients with moderate, or higher severity of the disease. RT-PCR assays were performed according to the protocol established by the WHO and previous studies. Each patient's nasopharyngeal and oropharyngeal swab samples were obtained and analyzed for SARS-CoV-2. The RNAs were extracted using the QIAamp™ viral RNA micro kit from Qiagen™ according to the manufacturer's instructions in a biosafety cabinet and in accordance with laboratory biosafety requirements. With E-gene and Rdrp-gene probe/primer and superscript™ III platinum, one-step qRT-PCR kit of Invitrogen company mixtures was prepared. The mixtures were transferred to Roche Light cycler™ 96 and Applied Biosystem ABI step one plus™ real time thermal cyclers with positive control and no template control (NTC) as well as an internal control [25, 27–30].

Patients eligible for admission were admitted in isolated wards and were visited daily by transplant and infectious disease specialists, who modified and adjusted their treatment regimens and immunosuppressive medications based on available international guidelines and underlying disease [23, 31–35]. Also, previously, some antivirals such as Lopinavir-Ritonavir, Hydroxychloroquine, and interferons were used to treat and manage these patients. But in time, as further studies disqualified their efficacy in COVID-19 treatment, they were replaced by other antivirals such as Remdesivir (5-day regimen). Clinical pharmacists were responsible for dose adjustments based on liver and kidney functions and also the consideration of drug-drug interactions.

In situations where at least 7–10 days had passed after the disease onset, according to the patient’s clinical status, the required amount of oxygen receives, the extent of pulmonary involvement, and the levels of inflammatory markers such as c-reactive protein (CRP), interleukine-6, or ferritin, the medical team decided to start using corticosteroids (Dexamethasone (6–8 mg/day) or Methyl Prednisolone (1 mg/kg)) or Tocilizumab (4–8 mg/kg, maximum dose: 800 mg). Discontinuation, decreasing the dose, or continuing to use an immunosuppressive regimen was based on the transplant team’s decision and the clinical status of the patients and their disease severity.

The criterion for receiving Remdesivir in our patients included moderate to severe or critical patients, either during the first 14 days of the initiation of COVID-19 related symptoms, in patients who have had symptoms for more than 14 days since the onset of symptoms, remdesivir was started only at the discretion of the infectious disease specialist and based on whether its benefits outweighed the risk. In patients with GFR below 30 mL/min, provided that the benefit outweighs the risk, remdesivir has been used with the same standard dose mentioned (200 mg loading dose then 100 mg daily up to 5 days), since as mentioned in several articles, remdesivir did not increase the risk of acute kidney injury (AKI) in patients with glomerular filtration rate (GFR) below 30 mLl/min [36, 37].

Also, Tocilizumab was administered in severe to critical patients, except for patients with neutrophil count < 500 cells/µL, platelets count < 50,000 µL, active tuberculosis, concomitant bacterial or fungal systemic infections, or if Alanine transaminase (ALT) or Aspartate transaminase (AST) were five times the upper limit of normal. Also, patients with clinical or laboratory signs of definite or suspected systemic or local infection, and who had high procalcitonin levels (> 2 ng/mL) were omitted from receiving tocilizumab with the opinion of an infectious disease specialist.

### Statistical analysis

Categorical variables were described as frequency rates and percentages, and continuous variables were described using mean, median, and interquartile range (IQR) or standard deviation (SD) values. Means for continuous variables were compared using the Mann–Whitney test. Proportions for categorical variables were compared using the χ^2^ test, and the Fisher exact test was used when the data were limited. All statistical analyses were performed using SPSS (Statistical Package for the Social Sciences), version 16.0 software (SPSS Inc). To evaluate the relationship between clinical outcomes and medications (or treatments), linear regression, and logistic regression were used. In the univariate model, *P* values were calculated and medications with a *P* value of lower than 0.25 were included in the multivariate model to determine significant medications. A *P* value of lower than 0.05 in the multivariate analysis was considered significant.

## Results

A total of 245 patients with a mean age of 49.59 (SD = 14.68, range: 21–77) years were included in our study. 52.24% of the patients were male and 47.76% were female. The mortality rate in our study was 8.16% (20 out of 245 patients). Also, 71 (52%) of patients with a disease severity from severe to critical were receiving dexamethasone and high dose methylprednisolone, while these amounts were 89 (87.25%) and 49 (81.6%) for patients receiving Remdesivir and Tocilizumab, respectively. The interval between drug administration from the onset of symptoms was 3.2 ± 5.1 days for remdesivir and 11.10 ± 2.90 days in the case of tocilizumab. Also, 81 patients received the combination of Remdesivir and Tocilizumab, and 5 patients received Remdesivir with high-dose corticosteroids. Table 1 shows the demographic data of the patients in our study.

**Table 1:** Demographic and clinical features of COVID-19 transplant patients including the association between the living and deceased groups (N = 245)

Variable	Total (%); *n* = *245*	Mortality (%)		*P* value*
		Alive; *n* = *225*	Death; *n* = *20*	
*Age group*				
< 40	109 (44)	97 (43)	12 (60)	0.97
40–60	87 (36)	84 (37)	3 (15)	
> 60	49 (20)	44 (20)	5 (25)	
*Gender*				
Male	128 (52)	120 (53)	8 (40)	0.70
Female	117 (48)	105 (47)	12 (60)	
*Comorbid diseases*				
Hypertension	97 (40)	92 (41)	9 (45)	0.17
Diabetes mellitus	25 (10)	18 (8)	7 (35)	< 0.01
Ischemic heart disease	67 (27)	60 (27)	7 (35)	0.01
Encephalopathy	2 (1)	2 (1)	0 (0)	1.00
Ascites	1 (0)	1 (0)	0 (0)	1.00
Deep venous thrombosis	1 (0)	1 (0)	0 (0)	1.00
Asthma	0 (0)	–	–	–
Chronic obstructive pulmonary disease	0 (0)	–	–	–
*Symptoms*				
Dyspnea	191 (78)	181 (80)	10 (50)	0.19
Fever	166 (68)	160 (71)	6 (30)	1.00
Malaise	107 (44)	99 (44)	8 (40)	1.00
Cough	91 (37)	90 (40)	1 (5)	0.27
Vomiting	145 (59)	141 (63)	4 (20)	0.68
Headache	88 (36)	88 (39)	0 (0)	0.34
Diarrhea	190 (78)	187 (83)	3 (15)	1.00
Chest pain	36 (15)	30 (13)	6 (30)	1.00
Rhinorrhea	14 (6)	14 (6)	0 (0)	1.00
Pharyngitis	0 (0)	–	–	–
*Severity*				
Moderate	178 (71.4)	174	4	
Severe	28 (11.4)	21	7	0.039
Critical	39 (15.9)	30	9	
*Transplantation status*				
Kidney transplant	143 (58)	134 (60)	9 (45)	0.44
Liver transplant	95 (39)	84 (37)	11 (55)	
Simultaneous pancreas-kidney	1 (0)	1 (0)	0 (0)	
Isolated bowel transplant	3 (1)	3 (1)	0 (0)	
Multivisceral transplant	3 (1)	3 (1)	0 (0)	
*Time after transplant (months)*				
1–3	69 (28)	63 (28)	6 (30)	0.021
3–6	111 (45)	110 (49)	1 (5)	
6–12	35 (14)	32 (14)	3 (15)	
> 12	30 (12)	20 (9)	10 (50)	
*Rejection*				
During hospitalization after COVID-19	22 (9)	20 (9)	2 (10)	0.22
*O*_*2*_* therapy*				
Nasal cannula	79 (32)	70 (31)	9 (45)	< 0.01
Mechanical ventilation	59 (24)	48 (21)	11 (55)	
None	107 (44)	107 (48)	0 (0)	
*Immunosuppressive medication*				
*Regimens*				0.29
Calcineurin inhibitors + Mycophenolate Mofetil	70 (29)	61 (27)	9 (45)	
Mycophenolate Mofetil + Prednisolone	29 (12)	25 (11)	4 (20)	
Calcineurin inhibitors + Prednisolone	142 (58)	136 (60)	6 (30)	
mTOR inhibitors	3 (1)	2 (1)	1 (5)	
*Changes*				0.17
No Change	101 (41)	99 (44)	2 (10)	
Decreasing the dose of Calcineurin inhibitors and Discontinue antimetabolite	90 (37)	88 (39)	2 (10)	
Discontinue all immunosuppressive	51 (21)	35 (16)	16 (80)	
Discontinue mTOR inhibitors	3 (1)	3 (1)	0 (0)	
*Treatment*				
Hydroxychloroquine	79 (32)	67 (30)	12 (60)	0.33
Lopinavir/ritonavir	90 (37)	71 (32)	19 (95)	< 0.01
Corticosteroid	136 (56)	125 (56)	11 (55)	0.06
Methylprednisolone	64 (26)	61 (27)	3 (15)	0.06
Dexamethasone	72 (29)	64 (28)	8 (40)	0.66
Interferon	55 (22)	46 (20)	9 (45)	0.34
Remdesivir	102 (42)	92 (41)	10 (50)	0.02
Tocilizumab	60 (24)	53 (24)	7 (35)	0.70

*Chi-square and Fisher exact tests

The mean length of intensive care unit (ICU) stay and hospital stay were 22.13 ± 11.01 and 33.19 ± 12.00 days, respectively. To evaluate the length of ICU and hospital stay among the patients and to make an accurate assumption, we excluded those who passed away during the course of hospitalization. Clinical outcomes and the effects of different therapeutic regimens in the treatment of COVID-19 in transplant patients are shown in Table 2. According to the results of this table, patients treated with Remdesivir have spent less time in the ICU and the hospital, compared with patients who have been treated with other antivirals (*P* value = 0.03). Also, patients treated with Tocilizumab spent fewer days in the ICU and the hospital, compared with patients who have been administered high doses of corticosteroids (*P* value = 0.04), however, there was no significant difference among the need for mechanical ventilation among the two groups (*P* value = 0.09). Based on multivariate regression, patients treated with Lopinavir/Ritonavir had an average higher length of stay in the ICU by 5.3 days (Table 3).

**Table 2:** Descriptive report of different treatment regimens’ effect on COVID-19 transplant patients' outcome

Drugs	Length of stay; mean ± SEM*		O_2_ therapy (%)		Mortality (%)	
	Intensive care unit stay	Hospital	Mechanical ventilation used	Mechanical ventilation not used	Yes	No
*Hydroxychloroquine*						
No	16.22 ± 9.87	27.21 ± 15.32	11 (19)	155 (83)	8 (40)	158 (70)
Yes	20.98 ± 10.09	30.87 ± 15	48 (81)	31 (17)	12 (60)	67 (30)
*Interferon*						
No	17.70 ± 15.44	26.59 ± 13.90	49 (83)	41 (48)	11 (55)	79 (35)
Yes	22.88 ± 16.79	31.11 ± 14.00	10 (17)	45 (52)	9 (45)	46 (20)
*Lopinavir/Ritonavir*						
No	20.19 + 16.60	24.40 ± 14.00	15 (25)	40 (47)	1 (5)	54 (24)
Yes	27.60 ± 15.43	34.30 ± 16.99	44 (75)	46 (53)	19 (95)	71 (32)
*Remdesivir*						
No	24.31 ± 12.41	34.45 ± 13.76	46 (78)	97 (52)	10 (50)	133 (59)
Yes	18.91 ± 10.00	27.00 ± 11.65	13 (22)	89 (48)	10 (50)	92 (41)
*Tocilizumab*						
No	23.30 ± 13.13	31.11 ± 12.00	49 (82)	136 (74)	13 (65)	172 (76)
Yes	18.83 ± 10.90	28.00 ± 14.10	11 (18)	49 (26)	7 (35)	53 (24)
*Dexamethasone or High dose methylprednisolone*						
No	24.33 ± 13.00	33.30 ± 13.00	45 (76)	64 (34)	9 (45)	100 (44)
Yes	19.77 ± 12.00	29.70 ± 11.10	14 (24)	122 (66)	11 (55)	125 (56)

*Standard error of the mean

**Table 3:** Effects of different treatment options on hospital and Intensive care unit stay of patients, modeled with univariate and multivariate linear regression

Drugs	Intensive Care Unit Stay				Hospital Stay			
	Univariate analysis		Multivariate analysis		Univariate analysis		Multivariate analysis	
	Coefficient (se*)	*P* value	Coefficient (se)	*P* value	Coefficient (se)	*P* value	Coefficient (se)	*P* value
Hydroxychloroquine	2.456 (1.44)	0.09	1.636 (1.02)	0.12	1.55 (2.21)	0.485	–	–
Interferon	3.97 (2.23)	0.08	0.759 (3.05)	0.80	1.39 (3.41)	0.685	–	–
Lopinavir/Ritonavir	7.83 (1.229)	< 0.001	5.309 (1.23)	< 0.001	3.27 (2.31)	0.161	3.44 (3.06)	0.27
Remdesivir	0.91	0.03	0.721(0.21)	0.03	− 0.77 (2.14)	0.01	0.10 (2.00)	< 0.001
High dose methylprednisolone or dexamethasone	0.103 (1.56)	0.94	–	–	1.572 (2.40)	0.514	–	–
Tocilizumab	0.71	0.02	0.66(0.10)	< 0.001	0.90 (1.10)	0.04	0.22	0.04

*Standard error

Another aim of our study was the evaluation of the relationship between mortality variables and different therapeutics. To evaluate the effect of corticosteroids versus other treatments, dexamethasone and high dose methylprednisolone (1 mg/kg) were combined as a single variable, labeled corticosteroid, and then, using multivariate logistic regression, we examined the effect of each drug on mortality (Table 4).

**Table 4:** Multivariate logistic regression analysis regarding the use of different therapeutic regimens in COVID-19 transplant patients and its association with mortality and need for mechanical ventilation (data are presented as odds ratio with 95% confidence interval)

Drug	Survival		Mechanical ventilation	
	OR (95% CI)	*P* value	OR (95% CI)	*P* value
Interferon	0.14 (0.02–1.94)	0.11	2.05 (0.33–12.66)	0.44
Hydroxychloroquine	0.87 (0.90–1.34)	0.49	1.19 (0.70–2.39)	0.20
Lopinavir/Ritonavir	0.14 (0.02–0.87)	0.04	3.22 (0.64–16.27)	0.16
Remdesivir	2.20 (0.07–0.81)	0.02	1.70 (0.85–4.40)	0.11
Corticosteroid (Dexamethasone and High dose methylprednisolone	0.33 (0.03–3.42)	0.35	4.59 (0.48–43.72)	0.19
Tocilizumab	1.10(0.05–0.92)	< 0.001	1.91 (0.07–1.10)	0.09

*OR* odds ratio; *CI* confidence interval

The results of Table 4 show that patients who were treated with Lopinavir/Ritonavir had a lower chance of survival (OR < 1; *P* value = 0.041) and those who were administered Remdesivir or Tocilizumab had a greater chance of survival (OR > 1; *P* value < 0.05). Also, none of the drugs had a significant association with the patients' need for mechanical ventilation. As shown in Table 5, clinical complications such as bacterial superinfections, elevation in liver enzymes, GFR reduction (< 30 mL/min), cardiovascular (QTc prolongation, arrhythmia, etc.) and gastrointestinal complications (diarrhea, vomiting, etc.) between different therapeutic regimens were not statistically significant during the hospitalization period.

**Table 5:** Frequency and percentage of clinical complications among solid organ transplant recipients regarding different therapeutic regimens during the hospitalization period

Medications	Complication					
	Bacterial superinfection; *n* = *58*	Elevated liver enzymes; *n* = *123*	Reduced glomerular filtration rate; *n* = *31*	Neutropenia or thrombocytopenia; *n* = *29*	Cardiovascular; *n* = *67*	Gastrointestinal; *n* = *93*
Hydroxychloroquine	10 (17)	32 (26)	3 (10)	2 (7)	31 (46)	49 (53)
Interferon	11 (19)	24 (20)	2 (6)	1 (3)	–	1 (1)
Lopinavir/Ritonavir	9 (16)	41 (33)	4 (13)	2 (7)	30 (45)	22 (24)
Remdesivir	7 (12)	11 (9)	12 (39)	4 (14)	–	9 (10)
Tocilizumab	6 (10)	8 (7)	6 (19)	13 (45)	2 (3)	7 (8)
Dexamethasone or High dose methylprednisolone	15 (26)	7 (6)	4 (13)	7 (24)	4 (6)	5 (5)
*P* value	0.09	0.11	0.88	1.32	1.09	0.77

## Discussion

Optimal management of COVID-19 in immunocompromised patients, particularly SOT recipients, poses a great challenge regarding the risk of more severe respiratory virus infection and higher rates of bacterial and fungal superinfections compared with their immunocompetent counterparts. The interactions between post-transplant-related medications with anti-viral drugs have made it more difficult to use the drugs commonly used in COVID-19 management. Moreover, clinical data regarding COVID-19 infection and its ideal treatment in the transplant population are limited. In this study, we aimed to evaluate different anti-viral and immunomodulatory regimens of COVID-19 treatment in transplant patients, since the declaration of the COVID-19 outbreak until now. Regarding the anti-viral regimens in our study, Lopinavir/Ritonavir showed a lower chance of patient survival with a higher hospitalization duration; while Remdesivir has decreased mortality rates and hospitalization periods in the hospital and ICUs.

In the early era of the COVID-19 pandemic, Lopinavir/Ritonavir was used to be prescribed commonly in patients infected with COVID-19, based on the results suggesting efficacy against another coronavirus. In our study, Lopinavir/Ritonavir not only increased the mortality rates but also notably prolonged the length of ICU stays, in which patients who took Lopinavir/Ritonavir stayed in ICU for 5.3 more days on average. Many studies reported similar results, discouraging the use of Lopinavir/Ritonavir in the transplant population. Circumventing the use of this drug is attributed to its adverse effects and interaction with immunosuppressants such as Tacrolimus and other medications used in the transplant population, such as fluoroquinolones to deal with Gram-negative infections. Furthermore, gastrointestinal upset (e.g., nausea and vomiting) is the most common adverse effect of this drug along with QT prolongation in combination with other drugs used in the COVID-19 treatment regimen. Moreover, a rise in liver enzymes was also reported in some cases. Concerning the reported effects, routine administration of Lopinavir/Ritonavir in transplant patients was discouraged.

After being approved by WHO in October 2020, Remdesivir use has been increased dramatically and multiple studies were published indicating its role in managing COVID-19 patients in the world, while there were also some studies reporting contrary efficacy results. Overall, there hasn’t been a conclusive study based on Remdesivir use in SOT COVID-19 patients up until now, while only some small or case-report studies on its efficacy in these patients have been published so far. For example, in a study by Duran et al., it has been suggested that early Remdesivir use in orthotopic heart transplant patients infected with SARS-CoV-2 would improve clinical outcomes in the patients. Our study confirmed that a 5-day regimen of Remdesivir will significantly decrease the mortality rates in transplant patients. Since this drug has no potential interactions with other immunosuppressive medications used in SOT patients' drug regimens, it seems that Remdesivir could be a promising anti-viral drug in managing transplanted COVID-19 patients. However, to establish a more precise conclusion, the results of larger multicenter studies with greater sample sizes should be analyzed. On the other hand, there is a concern about nephrotoxicity and elevation of hepatic enzymes occurrences after receiving Remdesivir, which is critical in decision making for liver or kidney transplant recipients. In a multi-center cohort study, it was reported that Remdesivir administration did not significantly increase the incidence of acute kidney injury, even in patients with GFRs lower than 30 mL/min. Additionally, in the "SIMPLE-Moderate trial" study (NCT04292730), a clinical trial to evaluate the efficacy and safety of a 5 or 10-day regimen of Remdesivir versus standard care, it was reported that hepatic enzyme elevation in the Remdesivir-receiving group was not higher than the control group. Our results suggest that a decrease in GFR or hepatic enzyme elevation is not significantly different between transplant patients who were treated with Remdesivir and patients treated with other anti-viral drugs. Similar results have also been reported in other studies performed on transplant patients. In conclusion, it seems that Remdesivir could be considered a promising anti-viral with appropriate efficacy and safety in transplant patients infected with SARS-CoV-2.

One of the challenging and controversial issues in managing COVID-19 among transplant patients is the use of immunomodulatory drugs. Pereira et al. confirmed the positive effect of high-dose steroid therapy in the case of lung transplantation after the first several days of the illness [38]. Liu et al. confirmed the beneficial use of corticosteroids in an infected case of a liver transplant, but they recommended low doses of steroids [39]. Zhu et al. also believed that appropriate doses of IV corticosteroids during a short period will not only protect the allograft from acute rejection, but also decrease the alveolar exudation and improve systemic symptoms due to its anti-inflammatory effects. On the other hand, several studies discouraged widespread use of corticosteroid medications, and even advised to discontinue or reduce the dose of steroids in transplant patients. They adduced to the potential negative effect of early corticosteroids administration in reducing pathogen clearance, while increasing viral shedding subsequent to immune response inhibition. Observational studies showed that corticosteroid treatment was linked to higher mortality rates and nosocomial infections for influenza and delayed virus clearance for SARS-CoV and Middle East respiratory syndrome coronavirus (MERS-CoV); however, there is limited data regarding SARS-CoV-2 [40]. Also, corticosteroids have been associated with an increased risk of bacterial and fungal superinfections [41–45].

Our results showed no significant improvement following the administration of high doses of corticosteroids based on the hospitalization period and mortality rates. Nevertheless, patients treated with high doses of corticosteroids experienced more episodes of bacterial superinfections, however not statistically significant.

One of the controversial issues of immunomodulator therapy is the administration of interleukin-6, such as Tocilizumab, in COVID-19 management. Earlier, extensive studies were conducted regarding using Tocilizumab in the management of COVID-19 patients, of which some showed promising results in decreasing mortality rates. Further, published results of several clinical trials regarding the evaluation of the efficacy of Tocilizumab, such as the COVINTOC study, showed that the combination of the drug with standard care has achieved higher success in decreasing the risks of requiring mechanical ventilation or death, compared to the combination of a placebo and standard care. The subjects were hospitalized due to COVID-19 pneumonia and did not receive mechanical ventilation when enrolled in the study. However, the TOCIVID study showed no difference between the two groups in the total risk of death by any cause [46].

Various studies have been conducted regarding the efficacy of Tocilizumab in transplant patients, each with different and contrary results. Some have reported lower mortality rates after using a combination of Tocilizumab with Hydroxychloroquine in COVID-19 patients who had received a kidney transplant or had received a liver transplant and required dialysis. However, retrospective studies have reported no significant decrease in mortality risk after using this drug, while also describing it as a safe drug to use for SOT patients.

Our results indicate that patients who were treated with Tocilizumab experienced a significantly lower duration of hospitalization duration in the hospital and ICUs and also lower mortality rates, however, without significantly affecting the need for mechanical ventilation. The risk of complication occurrences after drug administration was not higher than other therapy groups. The most probable reason behind diverse results between these studies is the basic differences in studies design; in which some studies were case reports, while some had a more extensive pool of patients. On the other hand, some of the studies, in the process of analyzing mortality rates among the groups who had not been treated with Remdesivir or Tocilizumab, have matched the patients based on critical mortality risk factors such as age, transplant type, and immunosuppressive regimen, while several considered this factor in their clinical trial. Also, there are differences in the onset of Tocilizumab administration and its enrolling requirements. In some studies, Tocilizumab was combined with high doses of corticosteroids and the drug administration had been started prior to intubation, while in some studies, the therapy had been started after patient intubation. Also, while some studies restricted mortality rates to its occurrence during hospitalization, several had followed up on the patients the mortality rates for 30 days after dismissing dates [47].

Our study had several limitations. The retrospective method may include the risk of having false-positive results as well as overestimation. Due to this, it seems that designing a clinical trial to analyze the results related to the administration of anti-viral and immunomodulator drugs in transplant patients is critical. Our study only includes the results during the hospitalization period, and the patients haven’t been followed up after being discharged. For example, 28-day mortality and long-term complications were not evaluated. Another limitation was that there was no control group including the non-transplant infected patients. Therefore, the unique features of the transplant patient in terms of clinical symptoms, response to medications, and potential adverse effects of drugs could not be measured. In this study, the primary goal was to study the clinical aspects of COVID-19 patients, and due to the limited resources and equipment of developing countries, molecular evaluation was very limited. We also didn’t evaluate more detailed virological features, such as characteristics of the viral genotype, the amount of the virus at the time of infection, and the change in the amount of the virus after treatment. Also, one of the factors influencing the reduction of mortality throughout the study timeline has been the more experienced medical staff in the field of management of patients with COVID-19, but it is somewhat difficult to assess the effect of this factor alone. Finally, in our study, some patients received multiple agents, and therefore the impact of each agent is difficult to tease out.

## Conclusion

Our data shows a meaningful decrease in mortality rates and hospitalization period amongst SOT patients after the administration of Remdesivir, as an anti-viral, and Tocilizumab, as an immunomodulator drug.

## Data Availability

SPSS data of the participant can be requested from the authors. Please write to the corresponding author if you are interested in such data.

## Abbreviations

AKI: Acute kidney injury
ALT: Alanine transaminase
AST: Aspartate transaminase
CI: Confidence interval
COVID-19: Coronavirus disease
CRP: C-reactive protein
GFR: Glomerular filtration rate
ICU: Intensive care unit
IQR: Interquartile range
MERS-CoV: Middle east respiratory syndrome coronavirus
OR: Odds ratio
RT-PCR: Real-time polymerase chain reaction
SARS-CoV-2: Severe acute respiratory syndrome coronavirus 2
SD: Standard deviation
SEM: Standard error of the mean
SE: Standard error
SOT: Solid-organ transplant
SPSS: Statistical package for the social sciences
WHO: World Health Organization

## References

[1] Alberici F, Delbarba E, Manenti C, Econimo L, Valerio F, Pola A, et al. Management of patients on dialysis and with kidney transplant during SARS-COV-2 (COVID-19) pandemic in Brescia. Italy Kidney Int Rep. 2020 Apr 4;5(5):580–85. 10.1016/j.ekir.2020.04.001 PMC7128395 32292866

[2] Saberian P, Mireskandari SM, Baratloo A, Hasani-Sharamin P, Babaniamansour S, Aliniagerdroudbari E (2020). Antibody Rapid Test Results in Emergency Medical Services Personnel during COVID-19 Pandemic; a Cross Sectional Study. Arch Acad Emerg Med.

[3] Sabetian G, Moghadami M, Hashemizadeh Fard Haghighi L, Shahriarirad R, Fallahi MJ, Asmarian N, Moeini YS. COVID-19 infection among healthcare workers: a cross-sectional study in southwest Iran. Virol J. 2021 Mar 17;18(1):58. 10.1186/s12985-021-01532-0 PMC7968574 33731169

[4] Shahriarirad R, Khodamoradi Z, Erfani A, Hosseinpour H, Ranjbar K, Emami Y (2020). Epidemiological and clinical features of 2019 novel coronavirus diseases (COVID-19) in the South of Iran. BMC Infect Dis.

[5] Ranjbar K, Moghadami M, Mirahmadizadeh A, Fallahi MJ, Khaloo V, Shahriarirad R (2021). Methylprednisolone or dexamethasone, which one is superior corticosteroid in the treatment of hospitalized COVID-19 patients: a triple-blinded randomized controlled trial. BMC Infect Dis.

[6] World Health Organization. WHO Coronavirus (COVID-19) Dashboard 2021. https://covid19.who.int/. Accessed 13 Aug 2021.

[7] Chi G, Lee JJ, Jamil A, Gunnam V, Najafi H, Memar Montazerin S (2020). Venous thromboembolism among hospitalized patients with COVID-19 undergoing thromboprophylaxis: a systematic review and meta-analysis. J Clin Med.

[8] Meshkat S, Salimi A, Joshaghanian A, Sedighi S, Sedighi S, Aghamollaii V (2020). Chronic neurological diseases and COVID-19: associations and considerations. Transl Neurosci.

[9] Sadeghi M, Saberian P, Hasani-Sharamin P, Dadashi F, Babaniamansour S, Aliniagerdroudbari E. The possible factors correlated with the higher risk of getting infected by COVID-19 in emergency medical technicians; A case-control study. Bull Emerg Trauma. 2021;9(2):67–72. 10.30476/BEAT.2021.89713 PMC8195834 34150916

[10] Shahriarirad R, Sarkari B (2020). COVID-19: clinical or laboratory diagnosis? A matter of debate. Trop Dr.

[11] Shahriarirad R, Fallahi M (2020). TB and the COVID-19 pandemic: brothers in arms against lung health. Int J Tuberc Lung Dis.

[12] Dashti AS, Ebrahimi K, Shahriarirad R, Ghotbabadi SH, Aminnia S. COVID-19 pandemic in the disguise of multi system inflammatory syndrome in children: a case series. Research Square. 2021.

[13] Fung M, Babik JM (2020). COVID-19 in immunocompromised hosts: What we know so far. Clin Infect Dis.

[14] Elias M, Pievani D, Randoux C, Louis K, Denis B, Delion A (2020). COVID-19 infection in kidney transplant recipients: disease incidence and clinical outcomes. J Am Soc Nephrol.

[15] Caillard S, Chavarot N, Francois H, Matignon M, Greze C, Kamar N (2021). Is COVID-19 infection more severe in kidney transplant recipients?. Am J Transplant.

[16] Mamode N, Ahmed Z, Jones G, Banga N, Motallebzadeh R, Tolley H (2021). Mortality rates in transplant recipients and transplantation candidates in a high-prevalence COVID-19 environment. Transplantation.

[17] Negahdaripour M, Shafiekhani M, Moezzi SMI, Amiri S, Rasekh S, Bagheri A (2021). Administration of COVID-19 vaccines in immunocompromised patients. Int Immunopharmacol.

[18] Rabinowich L, Grupper A, Baruch R, Ben-Yehoyada M, Halperin T, Turner D (2021). Low immunogenicity to SARS-CoV-2 vaccination among liver transplant recipients. J Hepatol.

[19] Boyarsky BJ, Werbel WA, Avery RK, Tobian AA, Massie AB, Segev DL (2021). Immunogenicity of a single dose of SARS-CoV-2 messenger RNA vaccine in solid organ transplant recipients. JAMA.

[20] Dharmapalan D, Jacob TJ (2021). COVID-19 eradication for vaccine equity in low income countries. Indian Pediatr.

[21] Askarian M, Erfani A, Taghrir MH (2021). Who should get the vaccine first? A glimpse at COVID-19 vaccination prioritization strategies. EXCLI J.

[22] Sabetian G, Shahriarirad S, Moghadami M, Asmarian N, Shahriarirad R, Askarian M, et al. High Post-infection protection after COVID-19 Among healthcare workers: a population-level observational study regarding SARS-CoV-2 reinfection, reactivation, and re-positivity and its severity. Research Square. 2021. 10.30476/IJMS.2023.97708.2951 PMC11053253 38680224

[23] Mirjalili M, Shafiekhani M, Vazin A (2020). Coronavirus disease 2019 (COVID-19) and transplantation: pharmacotherapeutic management of immunosuppression regimen. Ther Clin Risk Manag.

[24] Avery RK (2021). COVID-19 therapeutics for solid organ transplant recipients; 6 months into the pandemic: where are we now?. Transplantation.

[25] Shafiekhani M, Niknam T, Tara SA, et al. COVID-19 Versus Applied Infection Control Policies in a Major Transplant Center in Iran. Research Square. 2021. 10.1186/s12962-023-00427-x PMC9969367 36849978

[26] World Health Organization. Clinical management of COVID-19: interim guidance, 27 May 2020. Geneva: World Health Organization; 2020 2020. Contract No.: WHO/2019-nCoV/clinical/2020.5.

[27] World Health Organization (2020). Laboratory testing for coronavirus disease 2019 (COVID-19) in suspected human cases: interim guidance, 2 March 2020.

[28] World Health Organization (2020). Coronavirus disease (COVID-19) technical guidance: Laboratory testing for 2019-nCoV in humans.

[30] Corman VM, Landt O, Kaiser M, Molenkamp R, Meijer A, Chu DK (2020). Detection of 2019 novel coronavirus (2019-nCoV) by real-time RT-PCR. Eurosurveillance.

[30] Sabetian G, Moghadami M, Haghighi LHF, Shahriarirad R, Fallahi MJ, Asmarian N (2021). COVID-19 infection among healthcare workers: a cross-sectional study in southwest Iran. Virol J.

[31] Cravedi P, Mothi SS, Azzi Y, Haverly M, Farouk SS, Pérez-Sáez MJ (2020). COVID-19 and kidney transplantation: results from the TANGO international transplant consortium. Am J Transplant.

[32] Maggiore U, Abramowicz D, Crespo M, Mariat C, Mjoen G, Peruzzi L (2020). How should I manage immunosuppression in a kidney transplant patient with COVID-19? An ERA-EDTA DESCARTES expert opinion.

[33] Fix OK, Hameed B, Fontana RJ, Kwok RM, McGuire BM, Mulligan DC (2020). Clinical best practice advice for hepatology and liver transplant providers during the COVID-19 pandemic: AASLD expert panel consensus statement. Hepatology.

[34] World Health Organization (2020). Clinical management of COVID-19: interim guidance, 27 May 2020.

[35] Samavat S, Nafar M, Firozan A, Pourrezagholi F, Ahmadpoor P, Samadian F (2020). COVID-19 rapid guideline in kidney transplant recipients. Iran J Kidney Dis.

[36] Ackley TW, McManus D, Topal JE, Cicali B, Shah S (2020). A valid warning or clinical Lore: an evaluation of safety outcomes of Remdesivir in patients with impaired renal function from a multicenter matched cohort. Antimicrob Agents Chemother.

[37] Adamsick ML, Gandhi RG, Bidell MR, Elshaboury RH, Bhattacharyya RP, Kim AY (2020). Remdesivir in patients with acute or chronic kidney disease and COVID-19. J Am Soc Nephrol.

[38] Pereira MR, Mohan S, Cohen DJ, Husain SA, Dube GK, Ratner LE (2020). COVID-19 in solid organ transplant recipients: Initial report from the US epicenter. Am J Transplant.

[39] Liu B, Wang Y, Zhao Y, Shi H, Zeng F, Chen Z (2020). Successful treatment of severe COVID-19 pneumonia in a liver transplant recipient. Am J Transplant.

[40] Yang J-W, Yang L, Luo R-G, Xu J-F (2020). Corticosteroid administration for viral pneumonia: COVID-19 and beyond. Clin Microbiol Infect.

[41] Lee N, Leo Y-S, Cao B, Chan PK, Kyaw W, Uyeki TM (2015). Neuraminidase inhibitors, superinfection and corticosteroids affect survival of influenza patients. Eur Respir J.

[42] Brun-Buisson C, Richard J-CM, Mercat A, Thiébaut AC, Brochard L (2011). Early corticosteroids in severe influenza A/H1N1 pneumonia and acute respiratory distress syndrome. Am J Respir Crit Care Med.

[43] Martin-Loeches I, Lisboa T, Rhodes A, Moreno R, Silva E, Sprung C (2011). Use of early corticosteroid therapy on ICU admission in patients affected by severe pandemic (H1N1) v influenza A infection. Intensive Care Med.

[44] Kim S-H, Hong S-B, Yun S-C, Choi W-I, Ahn J-J, Lee YJ (2011). Corticosteroid treatment in critically ill patients with pandemic influenza A/H1N1 2009 infection: analytic strategy using propensity scores. Am J Respir Crit Care Med.

[45] Han K, Ma H, An X, Su Y, Chen J, Lian Z (2011). Early use of glucocorticoids was a risk factor for critical disease and death from pH1N1 infection. Clin Infect Dis.

[46] gov C. Tocilizumab in COVID‐19 pneumonia (TOCIVID‐19).

[47] Pereira MR, Aversa MM, Farr MA, Miko BA, Aaron JG, Mohan S (2020). Tocilizumab for severe COVID-19 in solid organ transplant recipients: a matched cohort study. Am J Transplant.

